# Phylogenetic and Spatiotemporal Analyses of Porcine Epidemic Diarrhea Virus in Guangxi, China during 2017–2022

**DOI:** 10.3390/ani13071215

**Published:** 2023-03-31

**Authors:** Jiaguo Bai, Chen Du, Ying Lu, Ruomu Wang, Xueli Su, Kechen Yu, Qiuying Qin, Ying Chen, Zuzhang Wei, Weijian Huang, Kang Ouyang

**Affiliations:** 1 Laboratory of Animal Infectious Diseases and Molecular Immunology, College of Animal Science and Technology, Guangxi University, Nanning 530005, China; 2 Guangxi Zhuang Autonomous Region Engineering Research Center of Veterinary Biologics, Nanning 530005, China; 3 Guangxi Key Laboratory of Animal Breeding, Disease Control and Prevention, Nanning 530005, China; 4 Key Laboratory of Prevention and Control for Animal Disease, Guangxi University, Nanning 530005, China

**Keywords:** porcine epidemic diarrhea virus, PEDV, S1 gene, phylogenetic analysis, spatiotemporal analysis, recombination

## Abstract

**Simple Summary:**

In this study, 673 diarrhea samples from 143 pig farms in Guangxi during 2017–2022 were collected and detected for PEDV. Ninety-eight strains were selected for S1 gene analyses and an increased number of strains in the G2c subgroup was found from 2019 onwards. Bayesian analysis revealed that Guigang may have been the epicenter of PEDVs in Guangxi. In addition, Guigang was identified as the primary hub from which PEDVs spread via two routes, namely Guigang–Wuzhou and Guigang–Laibin. Further recombination analyses indicated that two of the strains, 18-GXNN-6 and 19-GXBH-2, originated from intra-genogroup recombination. This study revealed a new status of PEDVs in Guangxi, China, which increases understanding of the prevalence, genetic characteristics and evolutionary profiles of the circulating PEDV strains in China.

**Abstract:**

Since 2010, porcine epidemic diarrhea virus (PEDV) has swept across China and spread throughout the country, causing huge economic losses. In this study, 673 diarrhea samples from 143 pig farms in Guangxi during 2017–2022 were collected and detected for PEDV. Ninety-eight strains were selected for S1 gene analyses and these strains were classified into four subgroups (G1b, G2a, G2b and G2c), accounting for 1.02 (1/98), 75.51 (74/98), 16.33 (16/98) and 7.14% (7/98) of the total, respectively. Importantly, an increased number of strains in the G2c subgroup was found from 2019 onwards. Bayesian analysis revealed that Guigang may have been the epicenter of PEDVs in Guangxi. In addition, Guigang was identified as the primary hub from which PEDVs spread via two routes, namely Guigang–Wuzhou and Guigang–Laibin. Moreover, several coinfections of novel PEDV variants bearing large deletions in the partial S1 protein and PEDVs possessing an intact partial S1 protein were found in pigs. Further recombination analyses indicated that two of the strains, 18-GXNN-6 and 19-GXBH-2, originated from intra-genogroup recombination. Together, our data revealed a new profile of PEDV in Guangxi, China, which enhances our understanding of the distribution, genetic characteristics and evolutionary profile of the circulating PEDV strains in China.

## 1. Introduction

Porcine epidemic diarrhea virus (PEDV) is a novel virus that causes severe enteric disease in pigs; it belongs to the genus Alphacoronavirus [1]. The spread of PEDVs in pigs occurs via the oral–fecal route as well as through feeding and aerosol transmission [2,3]. After a disease outbreak, PEDV will cause a watery diarrhea as well as dehydration and growth retardation. It can lead to 100% mortality in newborn piglets during their first 7 days of life when infected with the virulent PEDV strains [4,5].

The first reported porcine epidemic diarrhea (PED) outbreak occurred in 1971 in UK pig herds [6,7]. Before 2010, PEDV was effectively controlled through the use of the CV777 vaccine [8]. However, with the emergence of variant PEDV strains, serious disease epidemics have been observed in China since October 2010 [9,10]. In America, PED has spread rapidly following its first outbreak in May 2013, affecting more than seven million piglets [11]. By 2014, the highly virulent PEDV had spread to other countries in the Americas, including Canada, Mexico and Colombia, causing huge economic losses to the global pork industry [12].

PEDV has an enveloped single-stranded RNA genome that is approximately 28 kilobases (kb) long with a 5′ untranslated UTR region as well as a 3′ UTR [8]. The PEDV genome encodes two replicase polyproteins (ORFs 1a and 1b), four structural proteins (spike S, envelope E, membrane M and nucleocapsid N) and one accessory protein encoded by ORF3 [13,14]. According to the difference of the S gene, PEDV can be divided into two types: G1 and G2 [15]. The S gene is considered to be the most useful gene for revealing the genetic diversity of PEDV isolates and it plays an important role in the molecular epidemiology and in the genetic variations of PEDV field strains [16,17]. The S protein is a receptor-binding protein that plays a crucial part in mediating viral entry into host cells by binding with host receptors and determining their immunogenicity [18]. It is divided into S1 (amino acids (aa) 1–729) and S2 (aa 730–1387) subunits [19]. In addition, it is involved in at least four neutralizing epitopes such as CO-26 K equivalent (COE) domain (at position 499–638 aa) of the PEDV Brl/87 [20], SS2 (748–755 aa), SS6 (764–771 aa) [21] and 2C10 (1368–1374 aa) [22]. It is also known to induce the production of major neutralizing antibodies [23].

Although there have been many previous studies on the genetic analysis of PEDVs, most of these focused on the phylogenetic and recombination analyses of the S1 gene. There have been only a few studies defining the history of PEDVs and their spreading trajectories. To better understand the spread and genetic evolution of PEDV in Guangxi, China and to provide more targeted strategies for its future prevention and control, 673 pig samples were collected to analyze the prevalence of this virus in different regions. Then, the partial S1 gene from 98 representative PEDV-positive samples were sequenced and analyzed. We conducted the phylogenetic, recombination, temporal dynamics and phylogeographic analyses of the PEDV strains isolated in Guangxi during the years 2017–2022.

## 2. Materials and Methods

### 2.1. Detection of PEDVs in Guangxi, China between 2017 and 2022

A total of 673 samples were collected from 143 pig farms in Guangxi from January 2017 to March 2022. The swine feces and intestinal contents were homogenized in a mixture of 20% glycerol and phosphate-buffered saline (PBS). The suspension was vortexed and centrifuged at 3000× *g* for 5 min. The clarified supernatants was collected and stored at −80 °C for RNA extraction, as previously described [24].

Total RNA was extracted from the collected samples using a humoral virus DNA/RNA kit (Axygen Scientific, Union City, CA, USA) according to the manufacturer’s instructions. Reverse transcription was carried out by adding oligo dTs, a dNTP mixture and an M-MLV reverse transcriptase reagent (TaKaRa, Dalian, China). A pair of primers targeting the N gene of the virus was designed in order to detect the PEDVs. Polymerase chain reaction (PCR) was utilized for the detection of PEDVs, using Green Taq Mix (Vazyme Biotech, Nanjing, China). The expected size of the amplicons generated was 492 bp, using the specific primer pair of PEDV-NF (5′-GAAATAACCAGGGTCGTGGA-3′) and PEDV-NR (5′- GCTCACGAACAGCCACAT-TA -3′). PCR products were amplified with 35 cycles at 95 °C for 15 s, 56 °C for 30 s and 72 °C for 30 s, with a final elongation at 72 °C for 10 min. The partial S1 gene of PEDV was amplified by using the primer pair of PEDV-pS11F (5′-AACACGTCATCGTCAGAGGC-3′) and PEDV-pS11R (5′- CGGTT-GGAGGTAAAACAGC -3′), with an expected size of 1500 bp. PCR products were amplified with 35 cycles of at 95 °C for 15 s, 56 °C for 30 s and 72 °C for 90 s, with a final extension at 72 °C for 10 min.

### 2.2. Sequencing of the Partial S1 Genes

In this study, 98 representative PEDV-positive samples were selected for partial S1 gene sequence analysis based on their geographical and temporal distribution (Supplementary Table S1). PCRs were then performed and the products were purified using a gel extraction kit (OMEGA biotech, Doraville, GA, USA). The PCR products were characterized by Beijing Genomics Institute (Guangzhou, China) and Sangon Biotech (Shanghai, China).

### 2.3. Sequence Alignment and Phylogenetic Analyses

Partial S1 gene sequences from 98 PEDV-positive samples were assembled using SeqMan software. Information regarding the identified strains and the 101 PEDV reference strains is provided in Supplementary Table S1. The deduced nucleotide and amino acid sequences were aligned and analyzed by using Clustal W in DNAStar 7.0. A phylogenetic tree was constructed by using the 1000-replicate neighbor-joining method called inter in the MEGA X software and annotated with the Interactive Tree Of Life (iTOL) software, which is an online tool for displaying and annotating the phylogenetic trees [25].

### 2.4. Bayesian Temporal Dynamics Analysis

A total of 110 partial S1 gene sequences from Guangxi (including 12 strains obtained from the Genbank database shown in the Supplementary Table S1) were aligned by using the MEGA version 7.0 program [26]. A maximum likelihood tree was reconstructed using the IQ-TREE software, implemented by the TN + F + R3 model with 1000 bootstrap replicates [27]. To determine the temporal structure, the regressions of the root-to-peak genetic distances of these sequences were determined by using the Treetime package [28]. The divergence times of PEDV S1 strain in BEAST version 1.8.3 were derived using a Bayesian Markov chain Monte Carlo (MCMC) approach (with an uncorrelated relaxation clock model), a Bayesian skyline coalescent and a TIM + F + G4 substitution model [29,30]. Then, the MCMC was run in parallel on 3 chains with 200 million steps per chain and a burn-in of 10%. Convergence was visually confirmed for all parameters (ESS > 200) by Tracer version 1.7 [31]. A maximum clade credibility (MCC) tree was obtained by using Tree Annotator (part of BEAST version 1.8.3) and visualized via FigTree version 1.4.4 [32].

### 2.5. Bayesian Phylogeographic Analysis

In order to obtain the distribution information of PEDV in different regions of Guangxi, the spatial distribution patterns were reconstructed using a phylogenetic analysis in BEAST 1.8.3 [33]. An asymmetric Bayesian stochastic search variable selection (BSSVS) surrogate model was used to analyze dispersal across geographic locations [29]. Significant transitions were determined based on the combination of both Bayes factor (BF) ≥ 3 and a mean indicator of 0.5; BF ≥ 1000, 100 ≤ BF < 1000, 10 ≤ BF < 100 and 3 ≤ BF < 10 indicated decisive support, very strong support, strong support and statistically significant support, respectively [34]. The BF value was calculated by using SPREAD3 v 0.9.7. The MCMC simulations with 200 million steps were run on three separate Markov chains and samples were collected from every 20,000 steps. Greater than 200 for each parameter of ESS was required [35].

### 2.6. Genome Recombination Events Analysis

All sequences were aligned by the Recombination Detection Program 4 (RDP 4), with the window size of 200 bp and the *p*-value of 0.05. Similar sequences were used for alignment with automatic sequence masking, as suggested in the RDP manual. Recombination events are counted by seven algorithms, including RDP, GENECONY, bootscan, MaxChi, Chimaera, SiScan and 3Seq in RDP 4. Putative recombination events were considered significant only if supported by at least three of the above approaches.

## 3. Results

### 3.1. Prevalence of PEDV in Clinical Samples from Diarrheal Pigs

**Figure 1:**
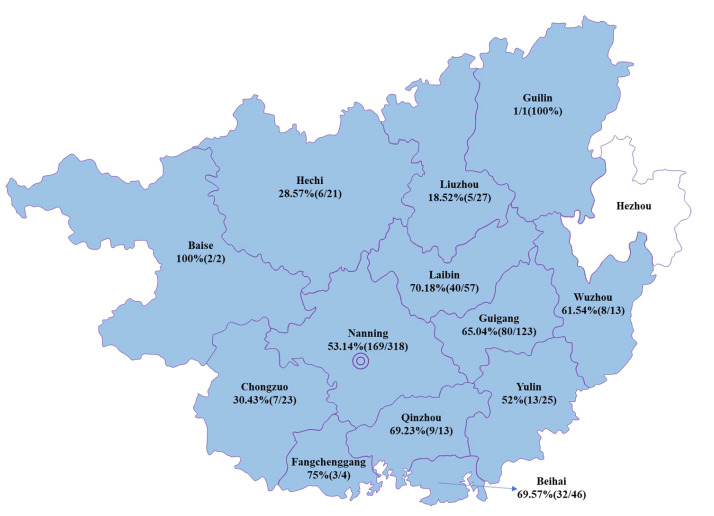
Map of PEDV distribution in pigs in 13 regions of Guangxi, China. The positive rates of PEDV infection are labeled for the regions. No clinical samples were collected from Hezhou.

RT-PCR results referring to the N gene fragment showed that 79.92% (110/143) of the sampled farms were positive for PEDVs, with an overall detection rate of 53.94% (363/673). From 2017 to 2022, the farm detection rate of PEDVs in Guangxi ranged from 50 to 89.29%. When the test results for the years 2017, 2018, 2019, 2020, 2021 and 2022 were counted separately, PEDV-positivity rates for the years were 59.12 (81/137), *71.*93 (123/171), 53.49 (23/43), 35.79, (34/95), 46.08 (94/204) and 34.78% (8/23), respectively (Table 1). PEDVs were detected in 13 regions of Guangxi, including the regions of Baise (100%), Guilin (100%), Fangchenggang (75%), Laibin (70.18%), Beihai (69.57%) and Wuzhou (61.54*%*), implying that these viruses existed widely in Guangxi (Figure 1).

### 3.2. Phylogenetic Analysis of the PEDV Partial S1 Gene

**Figure 2:**
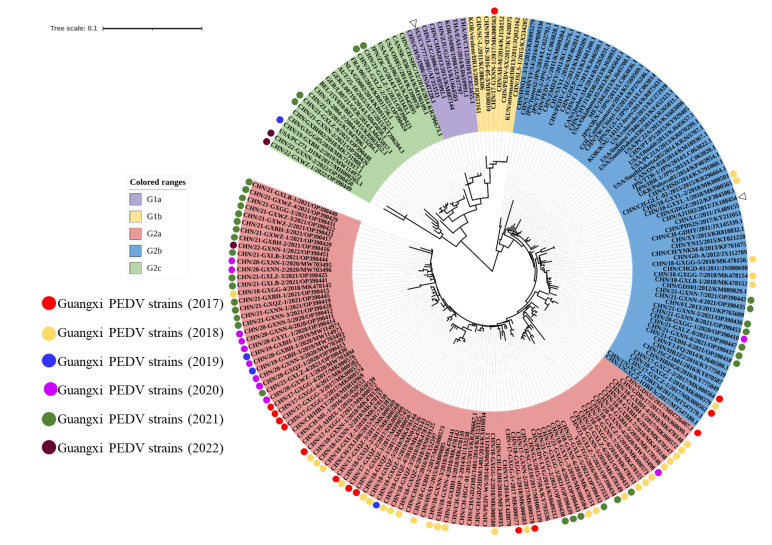
Phylogenetic analysis of the partial S1 gene sequences of 98 PEDV strains collected in this study, with 101 global reference strains. The tree was constructed using the neighbor-joining method for 1,000 replications in MEGA X software. Clades of different genogroups were colored as follows: G1-a (light purple), G1-b (light yellow), G2-a (light red), G2-b (light blue) and G2-c (light green). The strains reported in this study and the representative reference strains from each genogroup are highlighted in circles of different colors depending on the year and the hollow triangles.

In order to understand the phylogenetic relationship of the 98 representative S1 genes obtained in this study, we constructed a phylogenetic tree with 101 reference strains (Figure 2). All the strains were divided into two major distinct groups, namely a classical G1 group (G1a and G1b) and a variant G2 group (G2a, G2b and G2c). The G1a and G1b subgroups consisted of the prototype strains CV777 and DR13, respectively. In this study, only one strain (17-GXNN-1) was found clustered into G1b; 75.51% (74/98) of the strains obtained in this study were classified into the G2a subgroup, together with other reference strains reported in China. In addition, 16.33% (16/98) of the strains belonged to the G2b subgroup and shared 97.10–99.50% homology with the prototype strain AJ1102 (Table 2). Interestingly, 7.14% (7/98) of the strains were clustered in the G2c subgroup, whereas this subgroup was not detected before 2019. In 2021 and 2022, 4 out of 34 and 2 out of 3 strains detected were clustered into the G2c subgroup, respectively, which indicated that the proportion of natural recombination within the PEDVs was rapidly rising.

### 3.3. Bayesian Temporal Dynamics Analysis

**Figure 3:**
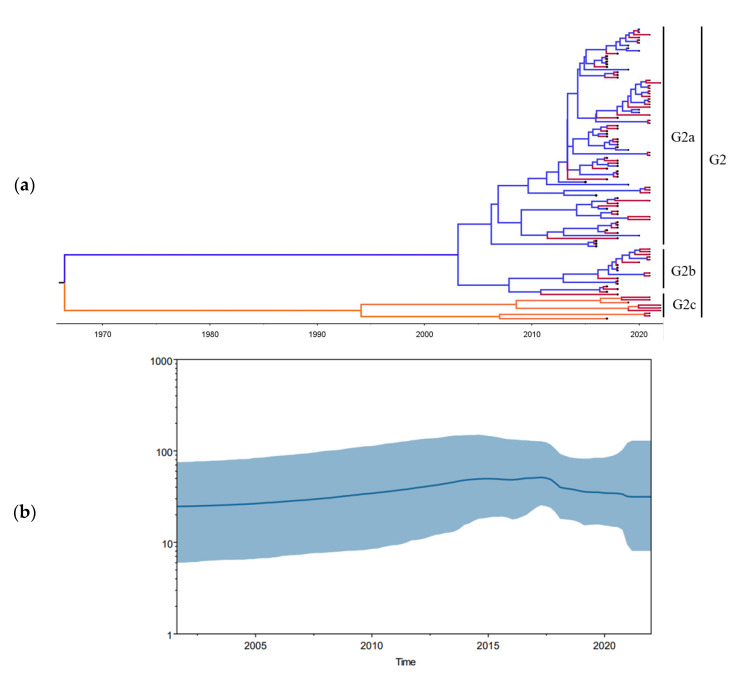
The maximum clade credibility (MCC) trees of the partial S1 gene sequences of PEDV strains found in Guangxi during 2015–2022. (**a**) The MCC tree was created using BEAST version 1.8.3 software; the Guangxi PEDV strains are marked with red nodes. Bayesian skyline plot of partial S1 gene sequences of PEDV strains. (**b**) Dark blue lines represent mean value of genetic diversity; light blue shading represents 95% confidence intervals.

The time-scaled MCC tree of PEDV strains based on the partial S1 gene sequences showed that all the PEDV strains were divided into four genotypes, and the results were consistent with the above phylogenetic analysis. Bayesian analysis identified the root of the tree in Guigang with a posterior probability of 0.94 (Figure 3a). The temporal changes in the effective population size of PEDV in Guangxi were shown in the Bayesian skyline plot (BKP) (Figure 3b). The population diversity of the virus had consistently maintained a slow growth since approximately 2002. By 2015, the effective population size of the virus continued to increase after a slight decrease and this showed an upward trend as a whole. The effective population size of the virus peaked in 2018 and then maintained a downward trend until 2022.

### 3.4. Bayesian Phylogeographic Analysis

**Figure 4:**
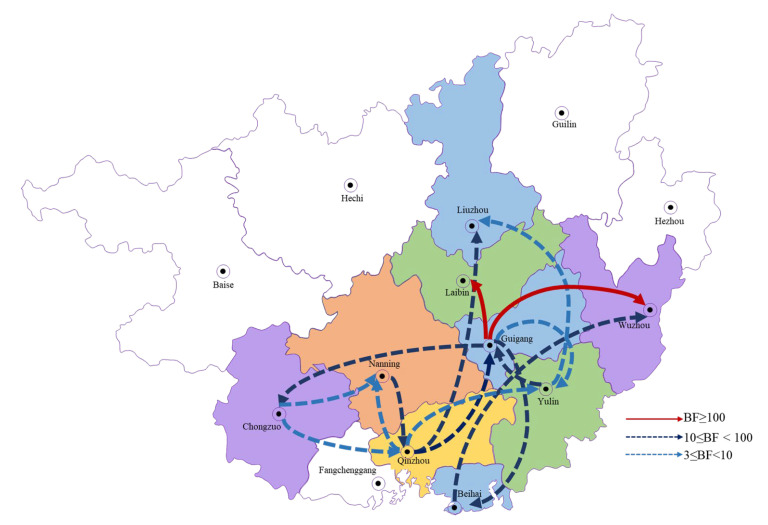
The dynamic analysis of the geographical regions of the partial S1 genes from Guangxi. The curve for all S1 partial genes in Guangxi shows the among-city virus lineage transitions statistically supported with a Bayes factor > 3. The solid red line represents BF ≥ 100 (indicated very strong support), the dark blue dotted line represents 10 ≤ BF < 100 (indicated strong support) and the light blue dotted line represents 3 ≤ BF < 10 (indicated statistically significant support).

Guangxi is divided into 14 regions and the sequences collected in this study came from 9 of these (namely Liuzhou, Laibin, Wuzhou, Guigang, Nanning, Chongzuo, Qinzhou, Yulin and Beihai). The spread circulation of PEDVs in Guangxi was estimated by BSSVS analysis. The 15 migration links in the spread of PEDVs in Guangxi were found by Bayesian phylogeographic analysis. Among them, Guigang–Laibin (BF ≥ 1000) and Guigang–Wuzhou (BF = 493) were identified with decisive support and very strong support, respectively. There were also seven other routes identified with strong support and six others were identified with indicated statistically significant support (Figure 4).

### 3.5. Deduence Analysis of the Partial S1 Protein

**Figure 5:**
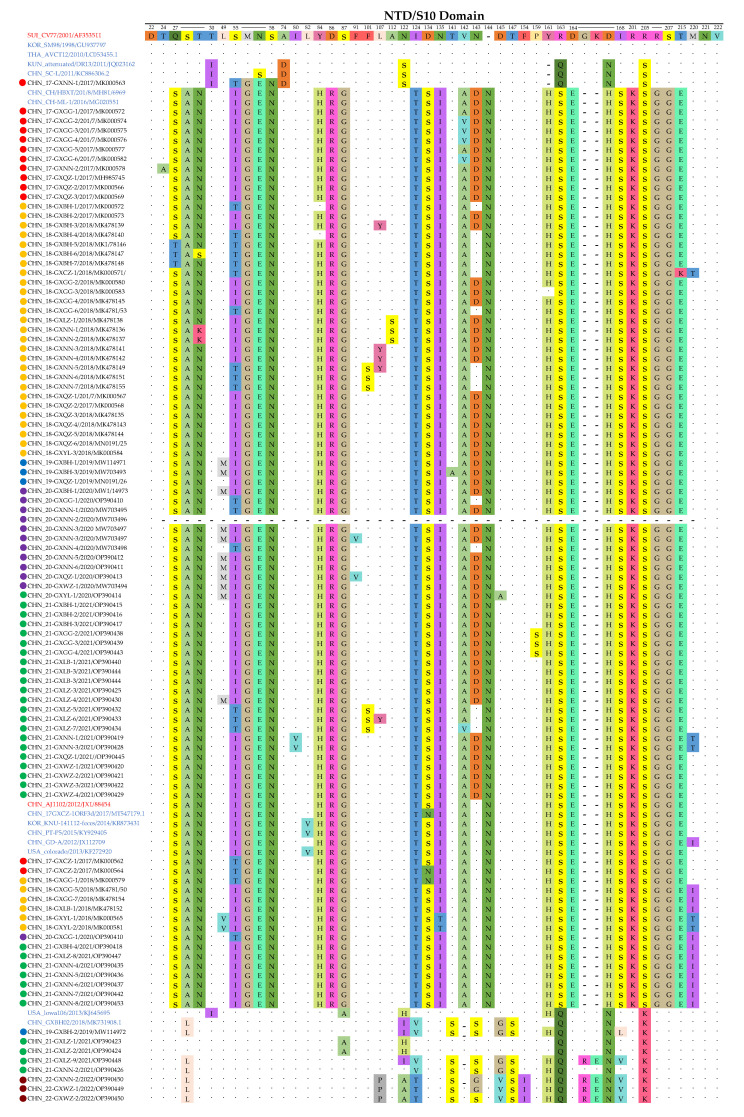
Amino acid variations in the neutralizing epitope regions of the partial S1 protein between the different subgroups. All the insertions, deletions and mutations in the epitope region, NTD/S10, are indicated. The vaccine strain CV777 (GenBank accession no. AF353511) was set as a reference; the two vaccine strains are highlighted in red, while the representative strains in each sub-group are colored in blue. The dots represent the consensus amino acids and the red boxes indicate the positions of insertions and deletions.

To elucidate the genetic identity of the detected strains between the subgroups, the NTD/S10 domain in the S1 gene was compared with the sequences of representative reference strains from each subgroup, including strains from G1a (CV777, SM98 and AVCT12), G1b (DR13 and SC-L), G2a (ML-1 and HBXT), G2b (AJ1102, 17GXCZ-ORF3d, KNU-141112-feces, PT-P5, GD-A and Colorado) and G2c (lowa106 and GXBH02) (Figure 5). Compared with CV777, the G2 group contained 15 distinct patterns of aa mutations (S28A, M56G, N57E, S58N, D86R, S87G, I124T, R163S, D164E, D167H, I168S, R201K, R205S, R206G and S207G), one additional 1aa insertion at position 143–144 (N) and two deletions at position 164–166. In addition, a 194aa deletion in the 20-GXNN-2 strain was found in this study.

**Figure 6:**
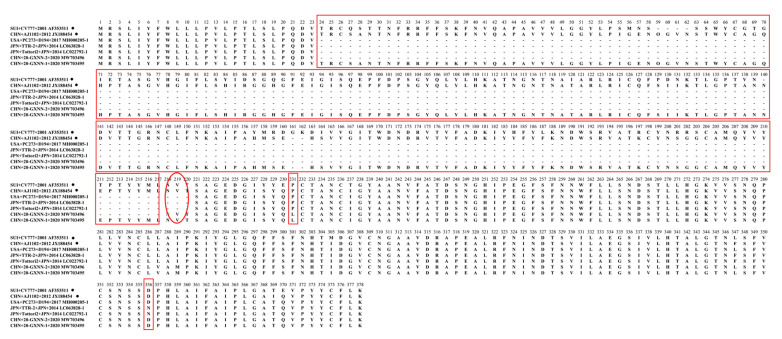
The alignment of amino acid sequences in the partial S1 protein of PEDVs in the Guangxi and reference strains. The deletions and mutations in the partial S1 protein of PEDV identified are shown in red boxes for the partial S1 protein.

To study the genetic characteristics of strain 20-GXNN-2, the partial S1 aa sequence was compared with other PEDV reference strains, including CV777, AJ1102, PC273/D194, TTR-2, Tottori2 and 20-GXNN-1 (Figure 6). The 20-GXNN-2 strain had a 194aa deletion at position 24–217 and a unique aa mutation (P231L) when compared with these classical strains. It was similar to the TTR-2/JPN/2014 strain (the first S1-NTD-del-PEDV variant strain identified in Japan in October 2014). Multiple amino acid alignments showed that the 20-GXNN-2 strain had the highest nucleotide identities (98.4%) with the 20-GXNN-1 strain collected from the same pig farm; it exhibited 95.6% amino acid homology when compared with the PC273/D194 strain, which contained similar deletions.

### 3.6. Recombination Analysis of the PEDV Partial S1 Gene

**Figure 7:**
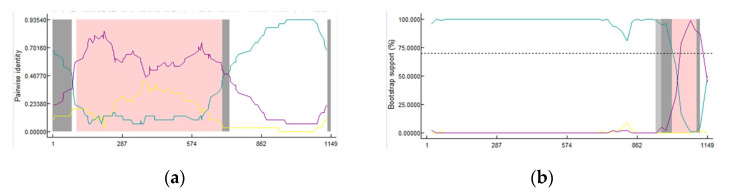
Detection of the potential recombination events in 19-GXBH-2 and 18-GXNN-6. Detection of potential recombination events in 19-GXBH-2: (**a**) RDP software-based screenshots showed the possibility of recombinant events among PEDV G2a PEDV-7C, PEDV G2c 19-GXBH-2 and the G1a strain SC-L; (**b**) detection of potential recombination events in 18-GXNN-6: bootscan screenshots showed the possibility of recombinant events among the PEDV G2a strains, CH/SCAZ10 and 18-GXNN-6, as well as the G1a strain CH/S.

Recombinant analyses were performed by using RDP4 software based on the PEDV partial S1 genes and the results revealed that there were two types of recombination events (Figure 7 and Table 3). The 19-GXBH-2 strain arose from recombination events between the PEDV-7C (G2a) and SC-L (G1a) strains and breakpoints for potential recombination were found in 54–703 nt (Figure 7a). The other strain, 18-GXNN-6, had a different type of recombination event (Figure 7b), where the zones out of the breakpoint 1004–1106 nt exhibited a greater similarity with CH/SCAZ10 (G2a). In addition, the zone between the breakpoint 1004–1106 nt had high levels of similarity with the CH/S (G1a) strain. These results suggest that strains 18-GXNN-6 and 19-GXBH-2 were the results of natural recombination events between the G2a and G1a subgroups.

## 4. Discussion

PEDV is a primary swine intestine pathogen that has given rise to serious economic losses for pig production and pork-associated businesses [36]. Vaccines for PEDV have been widely used in pig farms up until now [37,38]. However, CV777-based vaccines cannot offer complete protection from PEDV infection [39]. In this study, 673 samples were collected from 143 pig farms in Guangxi from January 2017 to March 2022. RT-PCR results showed that the prevalence of PEDVs in Guangxi diarrheal pigs was still high, with the total positive rate of 53.94% during 2017–2022. Interestingly, the prevalence of PEDVs has been steadily decreasing since 2019. The reason may be due to the outbreaks of African swine fever (ASF) that had forced farms to increase their awareness of biosecurity prevention and controls [40].

There have been several studies conducted on the occurrence of the PEDV virus in China and these studies have significant implications for understanding ongoing global PEDV outbreaks [41,42,43]. The Guangxi province, which is located near Southeast Asian countries, is a significant area for pig breeding in China. However, there is limited information available regarding the presence of PEDV in this region. In this study, the prevalence of PED and an analysis of the PEDV partial S1 gene sequence were conducted from samples obtained from Guangxi Province. This will increase our knowledge regarding the evolution of PEDVs and provide information on how to control its spread in the future.

PEDVs can be divided into two major groups, namely classical G1 (G1a, G1b) [41] and variant G2 (G2a, G2b and G2c) [44,45]. Here, we analyzed the representative 98 strains of the S1 partial gene; 1 strain named 17GXNN-2 was found clustered into G1b, which was more closely related to the classical PEDV strains, and the rest were all variant strains (74 in G2a, 16 in G2b and 7 in G2c). Notably, an increased number of strains in the G2c clade was detected and there were seven strains that showed greater correlation to the recombinant strains; these were grouped within the G2c clade. These results suggested that natural recombination may be a new variation trend of PEDVs in Guangxi.

In order to obtain a comprehensive understanding of the prevalence, evolution and spatial distribution of PEDV in Guangxi, a 5-year epidemiological analysis of 110 PEDV isolates from 2017 to 2022 was performed at the province level. The time-scaled MCC tree of PEDV strains showed that the root of the tree was in Guigang, with a high posterior probability, indicating that this region might have been the origin of PEDVs in Guangxi. BSP analysis showed that temporal changes occurred in the effective population size of the viruses. According to the population changes of PEDVs, it could be found that the virus maintained an upward trend until 2018. This may have been due to the appearance of large-scale epidemic PEDV mutants as well as the diminishing protective effects of vaccines that were based on the CV777 vaccine strains [46]. After 2018, the population size of the virus decreased rapidly, which may have been due to the use of a G2-based vaccine in Guangxi that effectively suppressed the spread of PEDVs.

In our study, Bayesian phylodynamic models were used to analyze the history of PEDVs in Guangxi; these served as a real-time early warning monitoring system to help farm managers to make correct decisions in order to reduce the risk of PED infection [18,38]. The Bayesian phylogeographic analysis showed that Chongzuo, Guigang and Beihai were the main import and export places of PEDVs in Guangxi and the two routes (from Guigang to Laibin and from Guigang to Wuzhou) were strongly identified with high BF values. It is understood that a large number of pigs had been shipped out from Guigang city every year, which is likely to be an important reason for why PEDVs had spread from there to other urban areas. In addition, the results of the Bayesian phylogeographic analysis revealed that there were several transmission chains of PEDVs in Guangxi, which were probably caused by the confusion of pig transportation. This emphasized that the designation of strict pig transportation policies would be crucial as a beneficial way to reduce the transmission chain of PEDVs and that this may be of great significance for the prevention and control of these viruses in Guangxi.

In addition to the accumulation of point mutations, another common pattern for the genetic evolution of coronaviruses is homologous recombination [15,47]. Two strains in this study might have been generated through intra-genogroup recombination determined by RDP analysis. The strain 19-GXBH-2 (G2c) arose from recombination events between the PEDV-7C (G2a) and SC-L (G1a) strains. In addition, breakpoints for the potential recombination were found in 54–703 nt, suggesting that the Guangxi PEDV strain may have evolved from a popular mutant strain; there may also be a recombination event in the partial sequence of the S1 gene. Whether these recombination events affect the virulence of the identified recombinants deserves further investigation. Intra-genogroup recombination provides a mechanism for amalgamation among these distinct subgroups and increases the gene pool of the co-circulating PEDVs.

Mutations, especially deletions and insertions in the S protein, may alter the virulence and tissue tropism of coronaviruses [48,49]. Similarly, variants of the PEDV S1 gene are considered to be responsible for viral antigenicity changes and these allow the viruses to escape the effectiveness of CV777-based vaccines [50]. The analysis of the partial S1 protein (NTD/S10 domain) indicated that the 20-GXNN-2 strain exhibited genetic signatures identical to the PC273/D194, TTR-2 and Tottori2 strains, including a 194-aa deletion (at positions 24~217) and a unique amino acid mutation (P231L), when compared with the CV777 and AJ1102 strains. Furthermore, a PEDV strain, JPN/Tottori2/2014, also had a 194 aa-deletion at positions 23–216 of the partial S1 protein. Interestingly, the strain PC177, with a 197 aa-deletion in a similar position (aa 34~230) of the partial S1 protein, was isolated from the Vero cell culture, indicating that a deletion at this position may affect the cellular adaptation and pathogenicity of PEDVs [49]. However, the 20-GXNN-1 strain that was collected from the same pig farm did not have these deletions and it showed an identical genetic signature to the vaccine strain AJ1102. These coexisting variants might be associated with the persistent PEDV infection in pig farms. Such a re-emerging and co-infection of the variants in this study have placed PEDV in a new situation, suggesting this disease has become more complex in terms of viral isolation, pathogenesis and epidemiology.

## 5. Conclusions

In conclusion, we collected 673 samples from 143 pig farms in Guangxi from January 2017 to March 2022. Ninety-eight strains were selected for S1 gene characterization and phylogenetic analysis and these showed that the prevalent PEDV strains in Guangxi were clustered into variant subgroups. These data indicate that Guigang was the epicenter of the dissemination of PEDVs in Guangxi. Bayesian phylogeographic analysis identified Guigang as the primary hub for the spread of PEDVs in Guangxi. In addition, amino acid alignment showed a 194aa deletion in the partial S1 gene of 20-GXNN-2 and recombinant analysis revealed two types of recombination events. This study show that enhanced epidemiological surveillance of PEDV in China is necessary. These results also provide more information about the prevalence and evolution of PEDVs and they will improve the monitoring and management of PEDV in Guangxi, as well as in other provinces in China.

## Figures and Tables

**Table 1:** The prevalence and genotypes of PEDVs in Guangxi during 2017–2022.

Prevalence of PEDV Infection			Genotypes of PEDV Strains (*n* = 98)			
**Year**	**Detection Rate**	**Positive Rate at Farm Level**	**G1b (*n* = 1)**	**G2a (*n* = 74)**	**G2b (*n* = 16)**	**G2c (*n* = 7)**
2017	59.12% (81/137)	89.29% (25/28)	1	11	2	0
2018	71.93% (123/171)	89.19% (33/37)	0	27	6	0
2019	53.49% (23/43)	60.00% (9/15)	0	3	0	1
2020	35.79% (34/95)	66.67% (12/18)	0	10	1	0
2021	46.08% (94/204)	71.79% (28/39)	0	22	7	4
2022	34.78% (8/23)	50% (3/6)	0	1	0	2
Total	53.94% (363/673)	79.92% (110/143)	1.02% (1/98)	75.51% (74/98)	16.33% (16/98)	7.14% (7/98)

**Table 2:** Homology analysis of the practical S1 gene of 98 PEDV strains collected in this study as well as 2 vaccine strains.

Guangxi PEDV Strains (n = 98)	Percentage of Nucleotide (Amino Acid) Identity (%)	
	CV777	AJ1102
G1b (*n* = 1)	95.10 (81.50)	93.50 (81.20)
G2a (*n* = 74)	85.30–91.50 (82.30–89.10)	96.40–99.50 (95.10–99.50)
G2b (*n* = 16)	85.30–86.40 (82.00–83.90)	97.10–99.50 (95.20–99.80)
G2c (*n* = 7)	87.40–91.60 (87.50–91.60)	84.30–87.10 (84.30–87.10)

**Table 3:** Characteristics of on the recombination events assessed.

Recombinant	Recombination Events	Putative Parental Virus		Detection Methods (Average *p*-Value)						
	Break Points ^†^	Major	Minor	RDP	GENECONV	Bootscan	Maxchi	Chimaera	SiSscan	3Seq
18-GXNN-6	1004–1106	CH/SCAZ10	CH/S	NS ^‡^	6.765 × 10^−1^	3.487 × 10^−5^	NS	NS	4.028	3.853
19-GXBH-2	54–703	PEDV-7C	SC-L	1.664 × 10^−6^	NS	NS	8.503 × 10^−3^	3.723 × 10^−2^	3.255 × 10^−27^	1.464 × 10^−4^

^†^: Break point of recombination events; ^‡^: No *p*-Value in detection methods.

## Data Availability

The partial S genome sequences of 98 PEDV strains obtained in this study have been deposited in the GenBank under the accession numbers MH985745, MK000562-MK000583, MK478135-MK478155, MN019126, MW114971-MW703498 and OP390413-OP390453, respectively.

## Supplementary Materials

The following supporting information can be downloaded at: www.mdpi.com/article/10.3390/ani13071215/s1, Table S1: Information regarding the determined strains in Guangxi during 2017–2022 and the reference strains of PEDV.
