# Supplementary material for: Phylogenetic and Spatiotemporal Analyses of Porcine Epidemic Diarrhea Virus in Guangxi, China during 2017–2022
Source: Animals (Basel). 2023 Mar 31;13(7):1215. doi: 10.3390/ani13071215 (PMC10093014; doi:10.3390/ani13071215)
Supplement: Supplementary file 1 [file animals-13-01215-s001.zip › animals-2287253-supplementary.pdf]

Supplementary Table S1. Information regarding the determined strains in Guangxi during 2017-2022 and the reference strains of PEDV.

Virus strain	Collection date	City of origin	Accession number	Virus strain	Collection date	Country of origin	Accession number
17-GXCZ-1	2017	Chongzuo	MK000562	CV777	2001	Belgium	AF353511.1
17-GXCZ-2	2017	Chongzuo	MK000564	JS-2004-2	2004	China	AY653204.1
17-GXGG-1	2017	Guigang	MK000570	LZC	2006	China	EF185992.1
17-GXGG-2	2017	Guigang	MK000574	virulent DR13	2009	South Korea	JQ023161.1
17-GXGG-3	2017	Guigang	MK000575	SM98	2010	South Korea	GU937797.1
17-GXGG-4	2017	Guigang	MK000576	CH/S	2011	China	JN547228.1
17-GXGG-5	2017	Guigang	MK000577	LC	2011	China	JX489155.1
17-GXGG-6	2017	Guigang	MK000582	SC-L	2011	China	KC886306.2
17-GXNN-1	2017	Nanning	MK000578	PEDV-7C	2011	China	KM609204.1
17-GXNN-2	2017	Nanning	MK000563	CH-GDHY-2011	2011	China	JX145339.1
17-GXQZ-1	2017	Qinzhou	MH985745	AJ1102	2011	China	JX188454.1
17-GXQZ-2	2017	Qinzhou	MK000566	attenuated DR13	2011	South Korea	JQ023162.1
17-GXQZ-3	2017	Qinzhou	MK000569	CHGD-01	2011	China	JN980698.1
18-GXBH-1	2018	Beihai	MK000572	CH/ZMDZY/11	2011	China	KC196276.1
18-GXBH-2	2018	Beihai	MK000573	AH-M	2011	China	KJ158152.1
18-GXBH-3	2018	Beihai	MK478139	CH/GDGZ/2012	2012	China	KF384500.1
18-GXBH-4	2018	Beihai	MK478140	GD-A	2012	China	JX112709.1
18-GXBH-5	2018	Beihai	MK478146	GDS01	2012	China	KM089829.1
18-GXBH-6	2018	Beihai	MK478147	OKN-1/JPN/2013	2013	Japan	LC063836.1
18-GXBH-7	2018	Beihai	MK478148	MEX/104/2013	2013	Mexico	KJ645708.1

18-GXCZ-1	2018	Chongzuo	MK000571	KGS-1/JPN/2013	2013	Japan	LC063814.1
18-GXGG-1	2018	Guigang	MK000579	IBR-1/JPN/2013	2013	Japan	LC063820.1
18-GXGG-2	2018	Guigang	MK000580	CH/YNKM-8/2013	2013	China	KF761675.1
18-GXGG-3	2018	Guigang	MK000583	CHM2013	2013	China	KM887144.1
18-GXGG-4	2018	Guigang	MK478145	CHYJ130330	2013	China	KJ020932.1
18-GXGG-5	2018	Guigang	MK478150	FJ-YX 2013	2013	China	KJ646590.1
18-GXGG-6	2018	Guigang	MK478153	FL2013	2013	China	KP765609.1
18-GXGG-7	2018	Guigang	MK478154	GDEP/2013	2013	China	KF601200.1
18-GXLB-1	2018	Laibin	MK478152	PC21A	2013	USA	KR078299.1
18-GXLZ-1	2018	Liuzhou	MK478138	PEDV-CHZ	2013	China	KM609209.1
18-GXNN-1	2018	Nanning	MK478136	USA/Colorado/2013	2013	USA	KF272920.1
18-GXNN-2	2018	Nanning	MK478137	USA/Indiana/17846/2013	2013	USA	KF452323.1
18-GXNN-3	2018	Nanning	MK478141	USA/Iowa106/2013	2013	USA	KJ645695.1
18-GXNN-4	2018	Nanning	MK478142	USA/Ohio60/2013	2013	USA	KJ645657.1
18-GXNN-5	2018	Nanning	MK478149	USA/Oklahoma35/2013	2013	USA	KJ645642.1
18-GXNN-6	2018	Nanning	MK478151	XY2013	2013	China	KR818832.1
18-GXNN-7	2018	Nanning	MK478155	YN15	2013	China	KT021228.1
18-GXQZ-1	2018	Qinzhou	MK478135	ZJU/G2/2013	2013	China	KU558701.1
18-GXQZ-2	2018	Qinzhou	MK478143	ON-018	2014	Canada	KM189367.1
18-GXQZ-3	2018	Qinzhou	MK478144	OH851	2014	USA	KJ399978.1
18-GXQZ-4	2018	Qinzhou	MN019125	NL/GD002/2014	2014	Netherlands	KR011122.1
18-GXQZ-5	2018	Qinzhou	MK000567	L00721/GER/2014	2014	Germany	LM645057.1
18-GXQZ-6	2018	Qinzhou	MK000568	KNU-141112-feces	2014	South Korea	KR873431.1
18-GXYL-1	2018	YuLin	MK000581	EAS1	2014	Thailand	KR610991.1

18-GXYL-2	2018	YuLin	MK000584	FJ-FQ 2014	2014	China	KJ646580.1
18-GXYL-3	2018	YuLin	MK000565	CHSD2014	2014	China	KX791060.1
19-GXBH-1	2019	Beihai	MW114971	CH-SDDP-3-2014	2014	China	KU133238.1
19-GXBH-2	2019	Beihai	MW114972	CH-SHC-12-2014	2014	China	KR296673.1
19-GXBH-3	2019	Beihai	MW703493	FR/001/2014	2014	France	KR011756.1
19-GXQZ-1	2019	Qinzhou	MN019126	COL/Cundinamarca/2014	2014	Colombia	KU569509.1
20-GXBH-1	2020	Beihai	MW114973	GER/L00719/2014	2014	Germany	LM645058.1
20-GXNN-1	2020	Nanning	MW703496	PEDV-WS	2014	China	KM609213.1
20-GXNN-2	2020	Nanning	MW703495	CAN/Quebec334/2014	2014	Canada	KR265831.1
20-GXNN-3	2020	Nanning	MW703497	PEDV-LYG	2014	China	KM609212.1
20-GXNN-4	2020	Nanning	MW703498	SK-030	2014	Canada	KM196109.1
20-GXNN-5	2020	Nanning	OP390411	Tottori2/JPN/2014	2014	Japan	LC022792.1
20-GXNN-6	2020	Nanning	OP390412	TTR-1/JPN/2014	2014	Japan	LC063822.1
20-GXGG-1	2020	Guigang	OP390410	TTR-2/JPN/2014	2014	Japan	LC063828.1
20-GXWZ-1	2020	Wuzhou	MW703494	TW-Chiayi-32	2014	China	KP276246.1
20-GXQZ-1	2020	Qinzhou	OP390413	Ukraine/Poltava01/2014	2014	Ukraine	KP403954.1
20-GXYL-1	2020	YuLin	OP390414	USA/SouthDakota285/2014	2014	USA	KR265787.1
21-GXBH-1	2021	Beihai	OP390415	USA/SouthDakota336/2014	2014	USA	KR265811.1
21-GXBH-2	2021	Beihai	OP390416	YC2014	2014	China	KT428879.1
21-GXBH-3	2021	Beihai	OP390417	ZK-O	2014	Japan	LC053343.1
21-GXBH-4	2021	Beihai	OP390418	CH/GDZH02/1401	2014	China	KR153325.1

21-GXGG-1	2021	Guigang	OP390431	CH/GDZHDM/1401	2014	China	KX016034.1
21-GXGG-2	2021	Guigang	OP390438	15V010/BEL/2015	2015	Belgium	KR003452.1
21-GXGG-3	2021	Guigang	OP390439	25-10_2015_AUT	2015	Austria	KT206204.1
21-GXGG-4	2021	Guigang	OP390443	CH/GX/2015/750A	2015	China	KY793536.1
21-GXLB-1	2021	Laibin	OP390440	AVCT12	2015	Thailand	LC053455.1
21-GXLB-2	2021	Laibin	OP390441	CH/HNYF/14	2015	China	KP890336.1
21-GXLB-3	2021	Laibin	OP390444	CH/JXJJ06/2015	2015	China	MG742381.1
21-GXLZ-1	2021	Liuzhou	OP390423	CH-GLC-02-2015	2015	China	KR296664.1
21-GXLZ-2	2021	Liuzhou	OP390424	CH-HGC-01-2015	2015	China	KR296667.1
21-GXLZ-3	2021	Liuzhou	OP390425	HNAY	2015	China	KR809885.1
21-GXLZ-4	2021	Liuzhou	OP390430	JSLS-1/2015	2015	China	KX534205.1
21-GXLZ-5	2021	Liuzhou	OP390432	ZL29	2015	China	KU847996.1
21-GXLZ-6	2021	Liuzhou	OP390433	CH/HN7/2016	2015	China	MF152597.1
21-GXLZ-7	2021	Liuzhou	OP390434	CH/HNQX-3/14	2015	China	KR095279.1
21-GXLZ-8	2021	Liuzhou	OP390447	PEDV-SX	2015	China	KY420075.1
21-GXLZ-9	2021	Liuzhou	OP390448	PT-P5	2015	China	KY929405.1
21-GXNN-1	2021	Nanning	OP390419	CH/SXYL/2016	2016	China	MF462814.1
21-GXNN-2	2021	Nanning	OP390426	CH-AHHX-1-2016	2016	China	MG020539.1
21-GXNN-3	2021	Nanning	OP390428	CH-ML-1-2016	2016	China	MG020551.1
21-GXNN-4	2021	Nanning	OP390435	HBHG6	2016	China	KY775049.1
21-GXNN-5	2021	Nanning	OP390436	HBJZ2	2016	China	KY775039.1
21-GXNN-6	2021	Nanning	OP390437	JLDH	2016	China	MF346935.1
21-GXNN-7	2021	Nanning	OP390442	JSCZ1601	2016	China	KY070587.1
21-GXNN-8	2021	Nanning	OP390453	PED-JS-2016-05-3	2016	China	MF038010.1

21-GXQZ-1	2021	Qinzhou	OP390445	TG5	2016	Viet Nam	MG373533.1
21-GXWZ-1	2021	Wuzhou	OP390420	PDS2S	2016	China	KY211053.1
21-GXWZ-2	2021	Wuzhou	OP390421	17GXCZ-1ORF3c	2017	China	MT547180.1
21-GXWZ-3	2021	Wuzhou	OP390422	17GXCZ-1ORF3d	2017	China	MT547179.1
21-GXWZ-4	2021	Wuzhou	OP390429	CH/SCAZ10/2017	2017	China	MH061339.1
22-GXNN-1	2022	Nanning	OP390449	GDS52	2017	China	MH726407.1
22-GXNN-2	2022	Nanning	OP390450	PC22A	2017	USA	KY499262.1
22-GXWZ-1	2022	Wuzhou	OP390451	PC273/D194	2017	USA	MH000205.1
22-GXWZ-2	2022	Wuzhou	OP390452	CH/HBXT/2018	2018	China	MH816969.1
				GXGG05	2018	China	MK731917.1
				GXBH02	2018	China	MK731908.1
				GDhy17	2019	China	MN368699.1
				CH/GX/2015/750A	2015	China	KY793536.1
				CH/GX/PEDV/1401/2016	2016	China	MZ364310.1
				CH/GX/PEDV/1008/2016	2016	China	MZ364309.1
				CH/GX/PEDV/997/2016	2016	China	MZ364308.1
				CH/GX/PEDV/938/2016	2016	China	MZ364307.1
				CH/GX/PEDV/1902/2017	2017	China	MZ364311.1
				CH/GX/PEDV/2373/2018	2018	China	MZ364314.1
				CH/GX/PEDV/1984/2018	2018	China	MZ364313.1
				CH/GX/PEDV/1939/2018	2018	China	MZ364312.1
				GX/HZ/CH/2018/Jan	2018	China	MK135453.1
				CH/GX/PEDV/2473/2019	2019	China	MZ364316.1
				CH/GX/PEDV/2467/2019	2019	China	MZ364315.1
